# Association between Androgenetic Alopecia and Psychosocial Disease Burden: A Cross-Sectional Survey among Polish Men

**DOI:** 10.1155/2022/1845044

**Published:** 2022-03-17

**Authors:** Roksana Adamowicz, Piotr Załęcki, Anna Dukiel, Danuta Nowicka

**Affiliations:** ^1^Faculty of Physiotherapy, Wroclaw University of Health and Sport Sciences, Wrocław 51612, Poland; ^2^Wroclaw Medical University, Wrocław 50367, Poland; ^3^Department of Dermatology, Venereology and Allergology, Wroclaw Medical University, Wrocław 50368, Poland

## Abstract

A decline in quality of life in men with androgenetic alopecia (AGA) is frequently reported, so we aimed to evaluate the psychosocial burden related to AGA in Polish male patients with AGA. We enrolled 75 adult patients with AGA. The study was conducted in an outpatient dermatology clinic in Poland. Each participant answered 23 dedicated questions about demographic data, history of the disease, and a psychosocial condition. Overall, 38.7% of patients did not notice any impact of AGA on their contact with other people or activity in their free time; 50.7% of patients observed little or no effect on their relationship with their partner; 60% of patients often or sometimes felt embarrassed by their baldness (mainly those aged 18–25 years; *p*=0.002); 66.7% of patients reported a rather large negative impact on their self-esteem; and 81.3% of patients sometimes experienced stress in everyday life. We conclude that AGA impairs the emotional condition and social functioning of men of all ages, but particularly of younger men. Broader interventions should be planned to allow access to a psychological and psychosocial support, starting treatment at an early stage of the disease, and involving family physicians in the treatment of AGA.

## 1. Introduction

Physical appearance is a significant factor in human identity. The first impression of the person is mainly focused on his/her face, including hair, which if looks good, suggests health and vitality. An image of a human being manifested by the body's appearance helps to stand out from the crowd and increases the self-assessment and mental health. Thick, shiny, and healthy hair adds to someone's appearance inflating the self-esteem.

Androgenetic alopecia (AGA) disturbs the good physical appearance. It is the most common form of alopecia. It is described as a progressive reduction in the diameter, length, and pigmentation of the hair. A multicenter study conducted in Europe, America, Africa, and Australia showed that AGA was the most frequent type of all 57 types of alopecia analyzed in a cohort of 2,835 alopecia patients with the share of 37.7%. The frequency and severity of AGA were higher in Caucasian men than in Asians and African Americans [1]. The disorder affects more men, up to 80%, than women, up to 50%, in the course of their life [2, 3]. Most patients observe the first signs of AGA before the age of 40 years, but there is a high percentage of people affected below the age of 30 years. The incidence of AGA increases with age [4].

Reports from the literature show that the occurrence of AGA is associated with the increased frequency of other diseases making it not only a cosmetic defect but also an important indicator of comorbidities. This is because many diseases share the same pathophysiology and/or are rooted in similar hormonal disorders. Hair thinning results from the effects of the testosterone metabolite dihydrotestosterone (DHT) on androgen-sensitive hair follicles. A male pattern is characterized by the bitemporal recession of the frontal hairline, followed by diffuse thinning at the vertex [5, 6]. Sanke et al. [7] showed the connection between the condition of hair in men and their hormonal status. They discovered that an early onset (before 30 years of age) of AGA in men with the phenotypic equivalent of the polycystic ovarian syndrome could be associated with an increased risk of developing other disorders such as metabolic syndrome, insulin resistance, obesity, cardiovascular disease, and infertility. Furthermore, they found many similarities in the hormonal profile between men with early-onset AGA and women with polycystic ovarian syndrome. Men with early-onset AGA presented with a significantly increased concentration of testosterone, dehydroepiandrosterone sulfate, and prolactin, while concentrations of follicle-stimulating hormone and sex hormone-binding globulin were significantly decreased. The insulin level and the grade of insulin resistance in men with AGA were similar to controls without AGA. Su et al. [8] attempted to link the presence of AGA in patients with diabetes mellitus and heart disease with the occurrence of premature death. The patients were followed for 57 months. The study showed that individuals with moderate-to-severe AGA vs no or mild AGA had a significantly higher risk of death from diabetes and heart disease after adjusting for age, family history of diabetes or heart disease, and metabolic status. The authors concluded that AGA would serve as an independent predictor of mortality from diabetes and heart disease. Another study by Polat et al. [9] suggested that the occurrence of AGA may be associated with a more frequent development of urolithiasis. The study suggested that patients with a vertex pattern AGA may have a 1.3 times higher risk of developing urolithiasis, while in men with total alopecia, this risk increases by 2.1 times. Nevertheless, the association of urolithiasis and elevated serum testosterone levels is still under debate in the literature studies.

Although the association of increased testosterone levels results in AGA and its health consequences in selected groups of patients burdened with chronic diseases, AGA has no direct impact on physical health or life expectancy. Yet, it results in a drastic change in appearance that can have a significant impact on mental health and quality of everyday life [10, 11]. A prospective, multicenter study on almost one thousand patients conducted by Han et al. [11] showed an important impact of AGA on the quality of life which was reduced to a greater extent in patients with severe alopecia, a longer duration of AGA, younger age, a history of previous nonmedical hair care, and past hospitalization for AGA. Similar outcomes were obtained among Polish men. [12] Interestingly, a recent study conducted by Lohia et al. [13] found that patients with AGA rate their hair loss as being more severe than their dermatologists. The outcomes of this study suggest that there is an unmet need among young patients with AGA regarding their psychosocial and quality of life which requires more attention from health care practitioners. Despite many researchers reporting a decline in quality of life and distress related to AGA in selected groups of patients, there is little evidence on the association between AGA and the psychosocial burden of this disease in the population of Polish men. [14–17] Particularly, a gap exists in the knowledge on psychosocial disease burden among Polish male patients treated in the private outpatient dermatologic clinics. Therefore, in this article, we aimed to evaluate the psychosocial burden related to AGA in the population of Polish male patients suffering from AGA.

## 2. Materials and Methods

We enrolled 75 adult patients with AGA of all ages. All men visited the clinic concerned about the condition of their hair and seeking advice on their hair problems. The study was conducted in a private outpatient dermatology clinic. The study was approved by the Senate Committee for Research Ethics at the University of Physical Education. Written informed consent was provided by all participants of this survey for answering the questionnaire.

After obtaining written informed consent for participation in the survey, patients were examined by an experienced dermatologist and completed a dedicated survey. The stage of the disease was rated by a dermatologist during the first or follow-up visit for the treatment for AGA using Norwood–Hamilton Baldness Scale [18, 19]. This scale is frequently used in clinical practice and clinical trials to evaluate the severity of hair loss. Controversies exist around its validity due to an unsatisfactory level of concordance and repeatability [19]. In this study, however, patients were examined only by one dermatologist which eliminates differences that might appear due to disagreements between researchers. Furthermore, the Norwood–Hamilton Baldness Scale has been widely used in clinical trials among Polish patients with AGA in order to classify the pattern of hair loss and to evaluate its severity [20]. Next, all men were asked to answer a 2-part questionnaire which comprised of 23 questions in total. The first part of the survey included the questions about demographic data, age of the onset of AGA, history of the disease, the use of special hair treatment and skincare, and past dermatological treatment. The second part of the questionnaire allowed for determining a link between AGA and patients' self-esteem at work and everyday functioning and social life and also their relation with loved ones. The questionnaire is included in Appendix 1. Participants were given a sufficient amount of time and were ensured full confidentiality while answering the questionnaire. All of them completed the survey by themselves. The questionnaires were filled anonymously.

The collected data were stored in the Microsoft Excel 2017 spreadsheet (Microsoft Corp., Redmond, WA, USA). Data were presented as counts and percentages or means with standard deviations. Comparisons were carried out using the Chi-squared test. All analyses were performed using R version 3.4.4 (R Foundation for Statistical Computing, Vienna, Austria).

## 3. Results

All invited men (*n* = 75) completed the questionnaire. The study group's mean age was 41 years; however, 34.67% (*n* = 26) were between the ages of 46 and 55 years. The patients were diagnosed with AGA Grade I–VII of the Hamilton–Norwood Scale. Over half of the study group (55.6%, *n* = 41) noticed first symptoms of AGA between the ages of 30 and 40 years. Rarely, they reported an early onset in men below 20 years of age (5.3%, *n* = 4). None reported a late onset in men above 50 years of age. Itching of the scalp was common among respondents; 9.3% (*n* = 7) reported it as a frequent symptom, 69.3% (*n* = 52) dealt with itching sometimes, and 21.3% (*n* = 16) has never experienced itching. The participants noted that AGA had an impact on their relationship with their partner, but it was not common. For most of them, AGA had little or no impact on their relationship with their partner and rarely caused discomfort in front of family and friends. Most study participants (50.7%, *n* = 38) never observed such an impact. Also, none of them observed it often. Among the remaining participants, 14.7% (*n* = 11) observed this impact sometimes, 21.3% (*n* = 16) rarely, while 13.3% (*n* = 10) were undecided. A few more participants felt discomfort in front of strangers. However, most of them (38.7%, *n* = 29) did not notice any AGA impact on their contact with other people or activity in their free time.

In the second part of the questionnaire, respondents reported the perceived impact of AGA on various domains. Most of the respondents sometimes felt embarrassed because of their disease. The younger men between the ages of 18 and 25 years had to cope with their embarrassment, most often than men in any other age range. The age groups differed significantly in terms of the frequency of being embarrassed. The respondents admitted that AGA had the biggest impact on their self-esteem in men aged 36–45 years. In every age group, participants affected with AGA struggled with stress on a daily basis. Most of them experienced stress often or sometimes, but neither of them denied that he was stressed. The most important domains of stress are presented in Table 1.

The frequency of being put in the stressful situation due to AGA differed depending on the place of living, but the difference among the groups was not statistically significant (Table 2).

Most participants (69.3%, *n* = 62) admitted that they were using supplements or other methods to cure their alopecia, 56.0% (*n* = 42) of them admitted that they undergo treatments aimed at curing excessive hair loss, and 30.7% (*n* = 28) of participants said that they never tried to cure this disease.

## 4. Discussion

Our study shows that AGA reduces the self-esteem of men of all ages, and it is a significant cause of embarrassment, particularly in younger patients. Although the topic of AGA has been widely discussed in literature, there is still a high percentage of men who have never tried to cure this condition. This situation is alarming because a number of treatments improving hair density and thickness as well as their growth are available. However, the awareness of using them among men could be insufficient. Moreover, the willingness of physicians to open a discussion on hair loss with their patients could be low.

AGA is a common, complex disorder that constitutes a therapeutic challenge. It coexists with hormonal disorders which may contribute to the development of chronic lifestyle diseases [7–9]. However, if AGA is diagnosed without comorbidities, it does not lead to disability, so the willingness of affected patients to treat AGA and treatment adherence are lower in comparison with other serious diseases [21]. These are further lowered by the low effectiveness of available therapies and their side effects. Shapiro et al. highlighted the complexity of the treatment of alopecias and provided a description of an organized diagnostic approach to support an accurate in-office diagnosis. They advised beginning the management with establishing an accurate diagnosis based on investigating medical and family history of concomitant illnesses and hormonal disorders, checking hormonal status, as well as establishing haircare practices and previous treatment of hair loss. A punch biopsy was mentioned to be useful in a differential diagnosis [22].

A patient with AGA can be offered a great variety of treatment modalities, including topical and hormonal treatment with oral finasteride and topical minoxidil solution having the good quality of evidence among male patients [23]. The effectiveness of oral finasteride varies depending on the clinical characteristics of patients from 32% to 80% [24, 25]. The short-term results are better than those obtained in the long-term treatment [26]. Topical minoxidil is considered more effective than systemic finasteride [24]. Other options include non-FDA-approved antihormonal treatments administered orally or applied topically, but their level of evidence is low. These are commonly used herbal medications [23]. Surgical treatment may be an option for patients who do not respond to other treatments but the effectiveness of hair transplants is still being investigated and requires support from hormonal treatment. Patients can also consider laser treatment [27].

Hair loss reduces the satisfaction of the body image, which affects particularly young men (between the ages of 26 and 35 years), as our study showed. Balding men can be considered less attractive in terms of their appearance and potential social functioning [28]. According to Zbiciak-Nylec et al. [17], men affected by AGA consider their baldness a factor that hampers starting relationships. More than half of them believe that people who do not suffer from hair loss are much more attractive. The disease burden put on people with AGA is a source of embarrassment. Rzepa et al. [16] revealed that the level of embarrassment due to AGA is 70% lower than that caused by AIDS or syphilis. Interestingly, patients with AGA considered onychomycosis and tuberculosis much less embarrassing than AGA. Conversely to our study, Sawant et al. [29] found younger men with AGA to be in significantly better psychological health with better self-assurance and greater acceptance of hair loss compared with older ones with AGA.

Embarrassing diseases lead to self-stigmatization. On the one hand, such patients limit their social activity considering themselves less attractive, but on the other hand, they may be considered by others as being less attractive [30, 31]. Perceived stigma in patients with alopecia is on a high level. Kacar et al. [32] compared patients with alopecia and mental disorder and found that almost all domains of the stigma scale were higher in patients with alopecia areata than in those with mental disorders. The systematic review of the literature conducted by Schielein et al. [33] shows that the evidence on stigmatization in people with AGA is very little. They report lower stigmatization in patients with alopecia areata than in those with AGA, but regardless of the type of alopecia and its etiology, patients with hair loss often suffer from stigmatization which in turn reduces their quality of life.

Lower quality of life in people suffering from AGA has been reported by many researchers. To assess the quality of life in dermatologic patients suffering from hair loss, the following 2 scales are used: Dermatology Life Quality Index (DLQI) [34] and the Hair-Specific Skindex-29 scale [35, 36]. Gupta et al. [37] confirmed the negative impact of AGA on the quality of life. They also reported a greater burden of the disease among younger men in comparison with older patients, which is in line with our findings. Patients from the study by Gupta et al. noted negative feelings when water gets in contact with the scalp and itching. They reported being humiliated for their alopecia. Russo et al. [38] found that a decline in quality of life-related to AGA was in the middle of the scale in comparison with other types of alopecia. It was higher than in patients with telogen effluvium but lower than in those with alopecia areata. Perceived anxiety was associated with a magnitude of a quality of life drop regardless of the type of alopecia. In a recent meta-analysis, Huang et al. [39] particularly focused on patients with AGA. They found those patients had a moderate impairment of health-related quality of life in general and in the emotion domain. Living in a relationship had a protective action against a decline in quality of life, while higher self-rated hair loss severity and higher educational level were associated with lower quality of life.

Several limitations must be taken into account when interpreting the results of our study. First, this study is a self-reported response survey, so the results could be affected by recall bias and social desirability. Second, we did not use a validated questionnaire which makes it impossible to compare the results of this study with the results of other researchers. Nevertheless, we tailored this questionnaire to the needs of the patients treated in our institution. Despite that, the results indicating lower self-esteem and emotional burden of AGA in younger men are in line with findings of other researchers. Finally, the population under study was limited to patients visiting one dermatology outpatient clinic and was relatively small. Patients visiting a dermatology clinic may be more aware focused on their health and more concerned about their appearance. For these reasons, the results should not be widely generalized.

## 5. Conclusions

Our study showed that AGA affects the emotional condition and social functioning of men of all ages, but particularly younger men. Treatment and counseling can play a role in leveraging self-esteem and quality of life in patients with AGA. Broader interventions should be planned to enable access to psychological and psychosocial support, starting treatment at an early stage of the disease and involving family physicians in the treatment of AGA.

## Figures and Tables

**Table 1:** Perceived symptoms due to androgenetic alopecia reported by the surveyed men.

	Years of age 18–25; *n* = 7	Years of age 26–35; *n* = 19	Years of age 36–45; *n* = 18	Years of age 46–55; *n* = 26	Years of age >55; *n* = 5	*p*-value
Embarrassment, *n* (%)						
Often	4 (57.1)	10 (52.6)	2 (11.1)	3 (11.5)	0 (0.0)	0.002
Sometimes	2 (28.6)	8 (42.1)	8 (44.4)	19 (73.1)	4 (80.0)	
Rarely	0 (0.0)	1(5.3)	7 (38.9)	3 (11.5)	0 (0.0)	
Never	1 (14.3)	0 (0.0)	1 (5.6)	1 (3.9)	1 (20.0)	
Effect on self-esteem, *n* (%)						
Big	1 (14.3)	5 (26.3)	9 (50.0)	10 (38.4)	1 (20.0)	0.637
Rather big	3 (42.8)	5 (26.3)	5 (27.8)	10 (38.4)	1 (20.0)	
Rather small	1 (14.3)	3 (15.8)	2 (11.1)	4 (15.4)	1 (20.0)	
Small	2 (28.6)	6 (31.6)	2 (11.1)	2 (7.8)	2 (40.0)	
Stress in daily life, *n* (%)						
Often	2 (28.6)	9 (47.4)	4 (22.2)	10 (38.4)	2 (40.0)	0.461
Sometimes	2 (28.6)	8 (42.1)	12 (66.7)	12 (46.2)	2 (40.0)	
Rarely	3 (42.8)	2 (10.5)	2 (11.1)	4 (15.4)	1 (20.0)	
Never	0 (0.0)	0 (0.0)	0 (0.0)	0 (0.0)	0 (0.0)	

**Table 2:** Perceived stress due to androgenetic alopecia reported by the surveyed men by the place of living.

	Village *n* = 24	City <5,000 inhabitants *n* = 12	City <50,000 inhabitants *n* = 9	City <250,000 inhabitants; *n* = 10	City >250,000 inhabitants; *n* = 20	*p*-value
Perceived stress, *n* (%)						
Often	9 (37.5)	5 (41.7)	1 (11.1)	5 (50.0)	7 (35.0)	0.125
Sometimes	12 (50.0)	2 (16.6)	7 (77.8)	4 (40.0)	11 (55.0)	
Rarely	3 (12.5)	5 (41.7)	1 (11.1)	1 (10.0)	2 (10.0)	
Never	0 (0.0)	0 (0.0)	0 (0.0)	0 (0.0)	0 (0.0)	

## Data Availability

The data used to support the findings of this study are available from the corresponding author upon request.

## Supplementary Materials

Supplementary Materials Appendix 1. Questionnaire.
