# Supplementary material for: Association between Androgenetic Alopecia and Psychosocial Disease Burden: A Cross-Sectional Survey among Polish Men
Source: Dermatol Res Pract. 2022 Mar 17;2022:1845044. doi: 10.1155/2022/1845044 (PMC8947924; doi:10.1155/2022/1845044)
Supplement: Supplementary Materials — Appendix 1. Questionnaire. [file 1845044.f1.docx]

Appendix 1. Questionnaire

1. What is your age range?


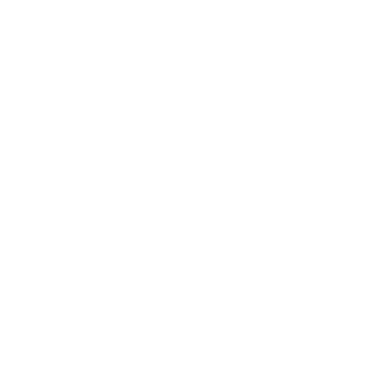
 18-25 years;
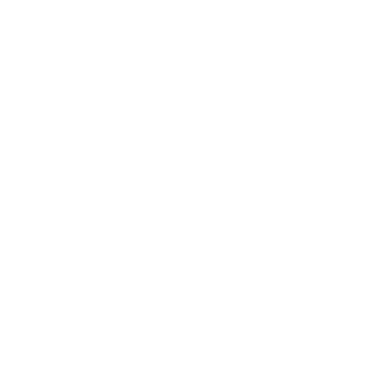
 26-35 years;
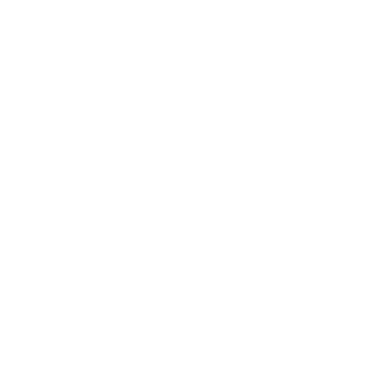
 36-45 years;
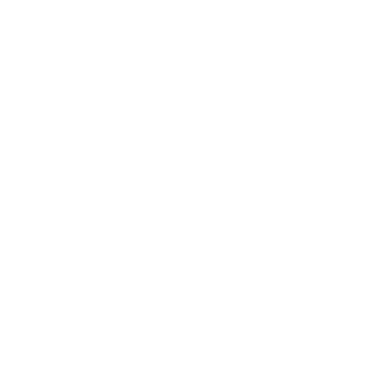
 46-55 years;
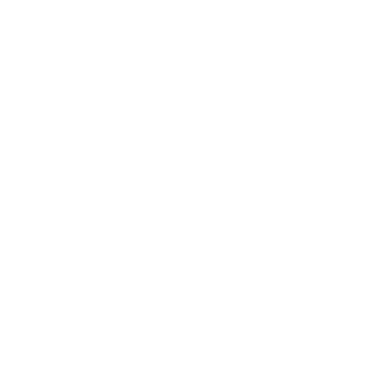
 over 55 years.

2. How big is the city you live in?


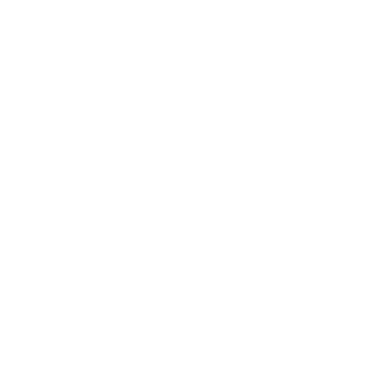
 Village;
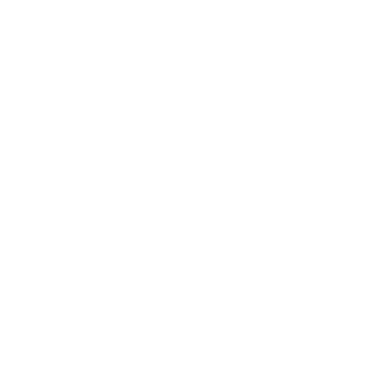
 City > 50.000 inhabitants;
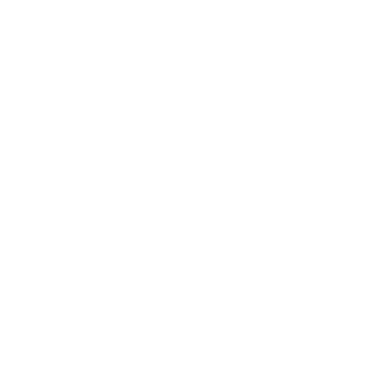
 City <50.000 and >100.000 inhabitants;
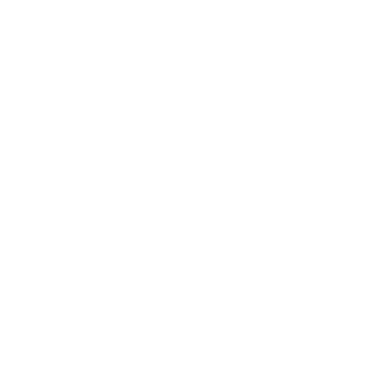
 City < 100.000 and >250.000 inhabitants;
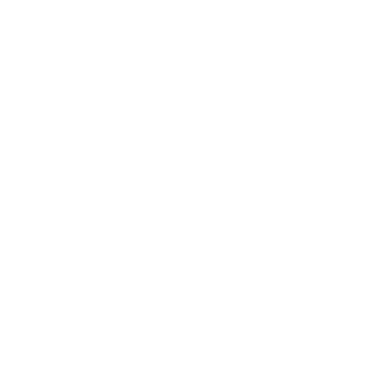
 City > 250.000 inhabitants.

3. What is your education?


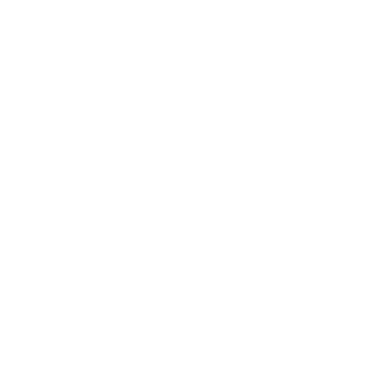
 Primary;
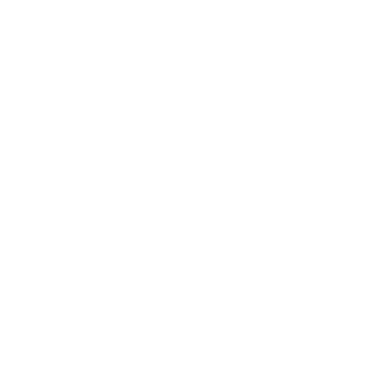
 Lower secondary education;
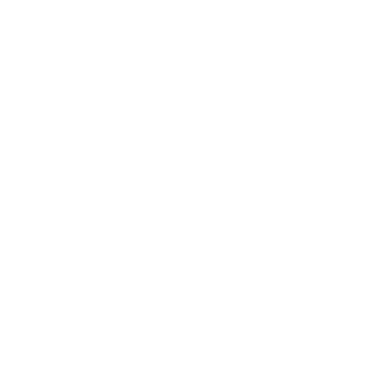
 Vocational;
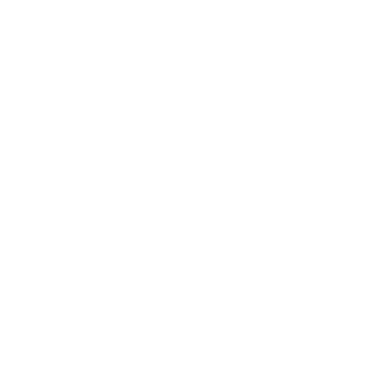
 Secondary;
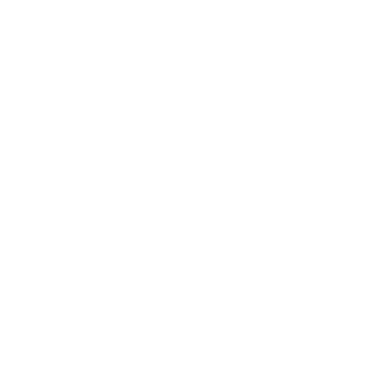
 University.

4. When did you noticed the first signs of alopecia?


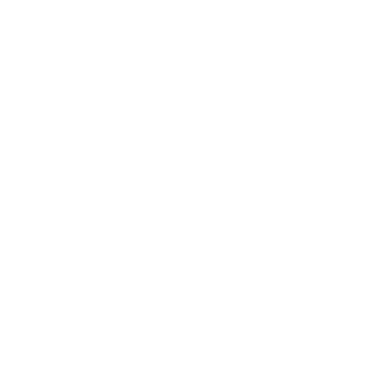
 before 20 years of age;
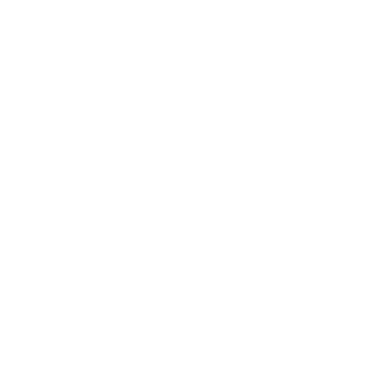
 20-30 years;
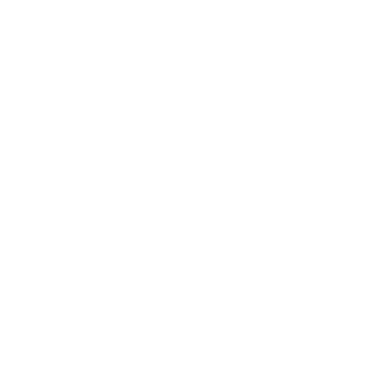
 31-40 years;
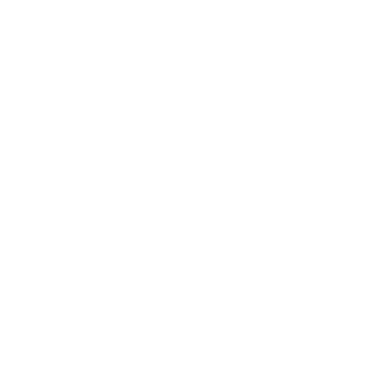
 41-50 years;
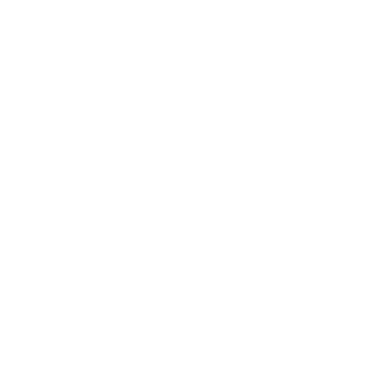
 over 50 years of age.

5. What was your hair density before androgenetic alopecia?


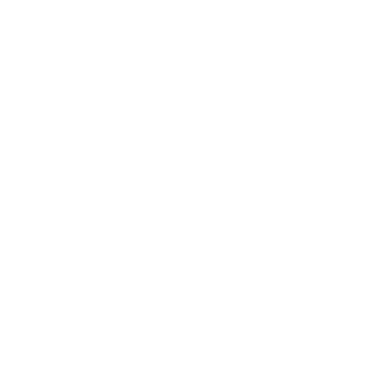
 High;
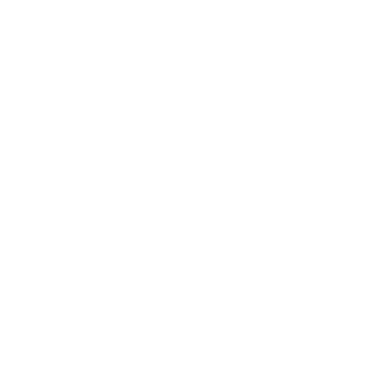
 Low;
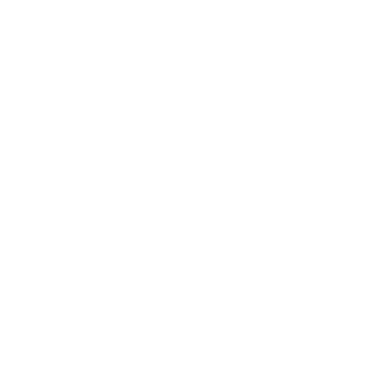
 Hard to say.

6. Did you notice androgenic alopecia in other members of your family?


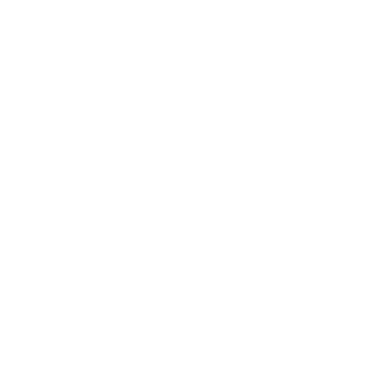
 Yes
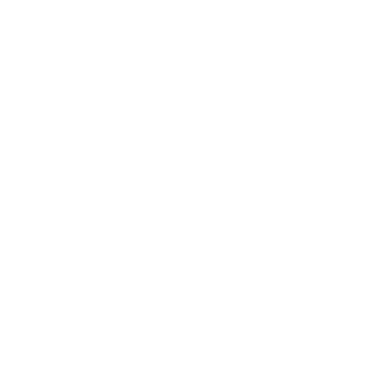
 No;
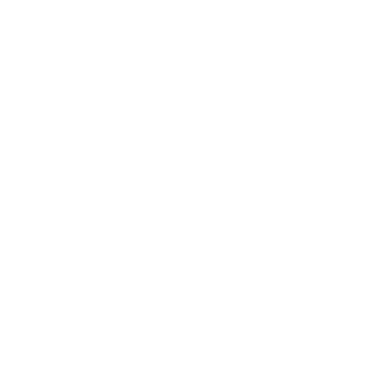
 I don’t know.

7. How often do you experience itching, tingling and sore scalp?


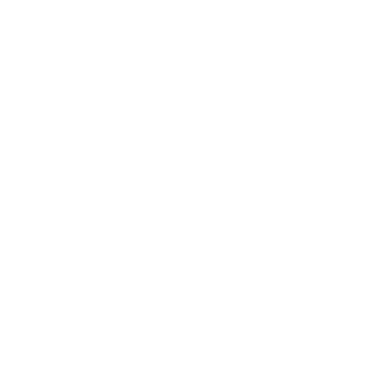
 Often;
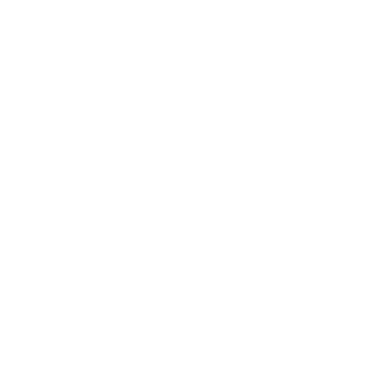
 Sometimes;
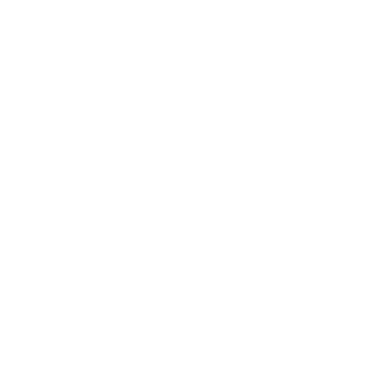
 Never.

8. How often are you exposed to stress in your everyday life?


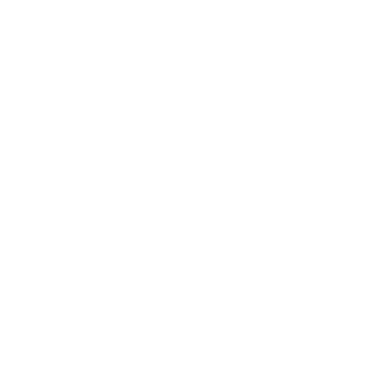
 Often;
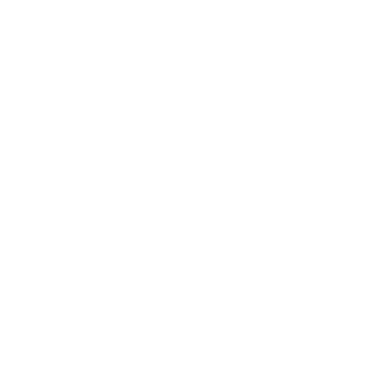
 Sometimes;
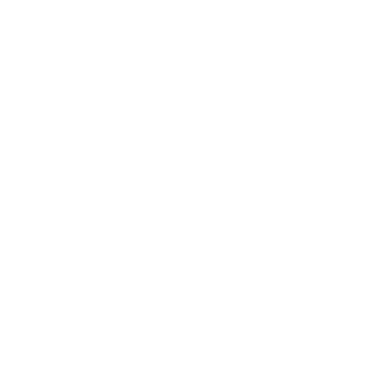
 Rarely;
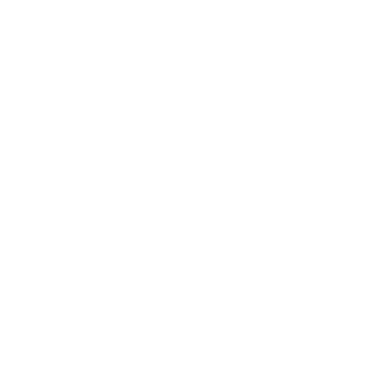
 Never.

9. How often do you feel embarrassed by your androgenetic alopecia?


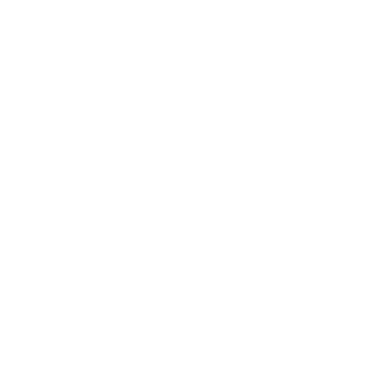
 Often;
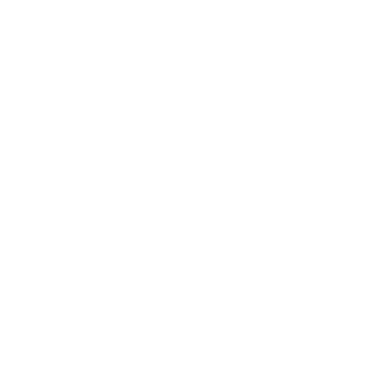
 Sometimes;
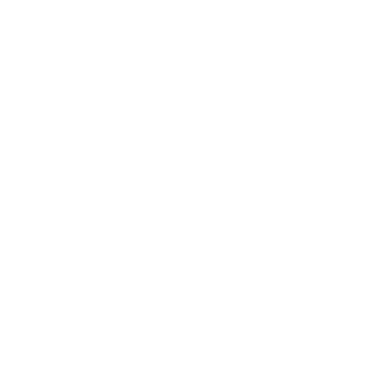
 Rarely;
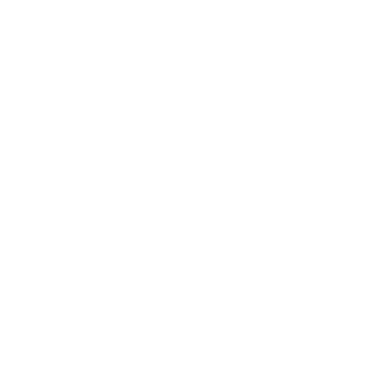
 Never.

10. Do you feel any discomfort caused by excessive hair loss in the presence of your family?


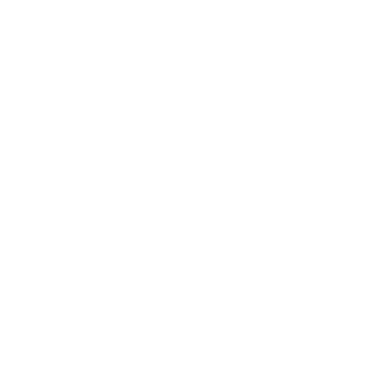
 To a great extent;
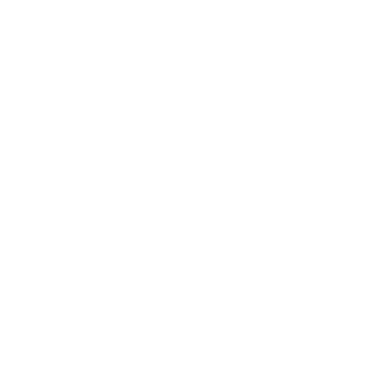
 Somewhat yes;
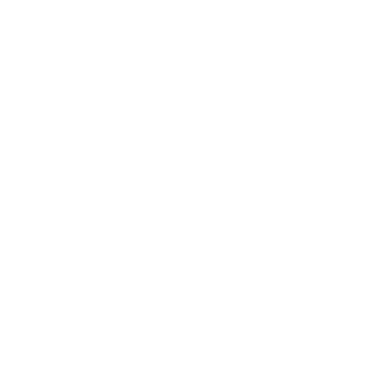
 Undecided;
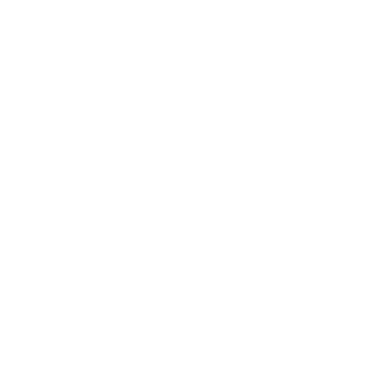
 Very little;
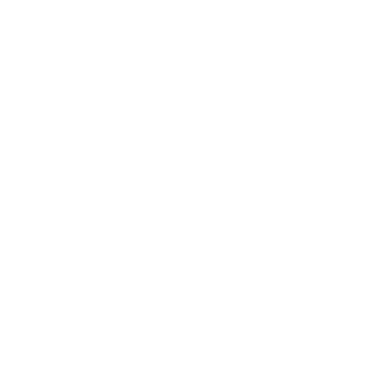
 Not at all.

11. Do you feel any discomfort caused by excessive hair loss in the presence of friends?


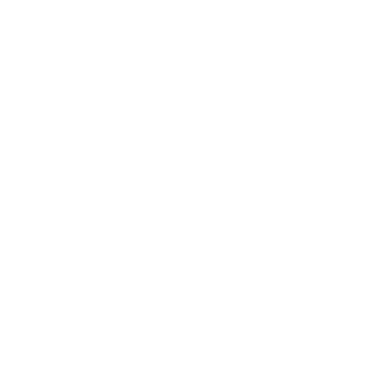
 To a great extent;
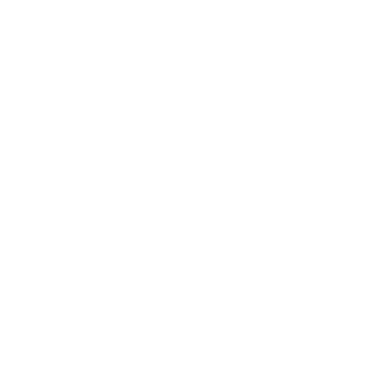
 Somewhat yes;
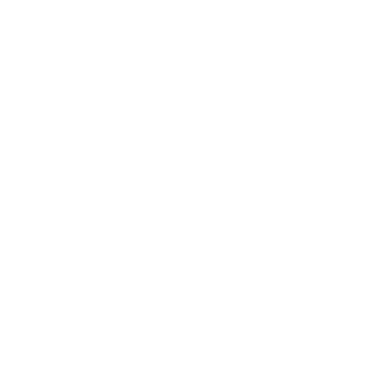
 Undecided;
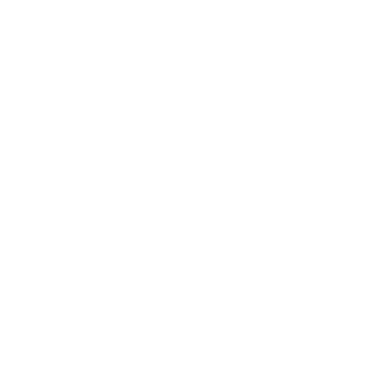
 Very little;
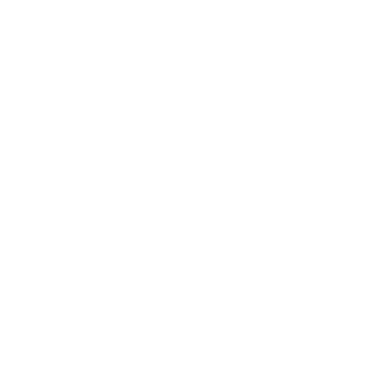
 Not at all.

12. Do you feel any discomfort caused by excessive hair loss in the presence of strangers?


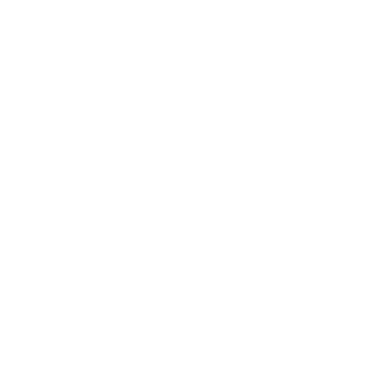
 To a great extent;
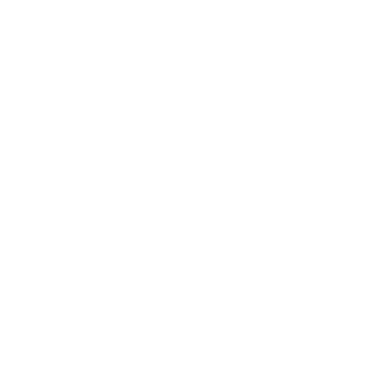
 Somewhat yes;
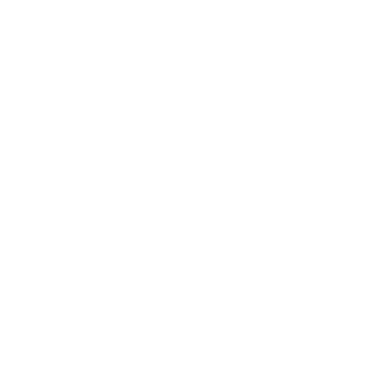
 Undecided;
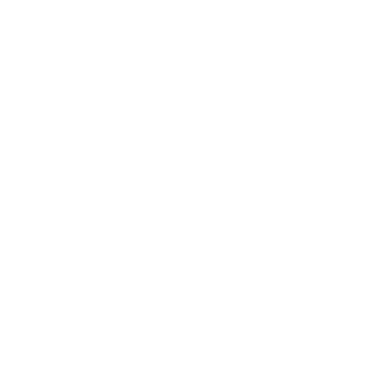
 Very little;
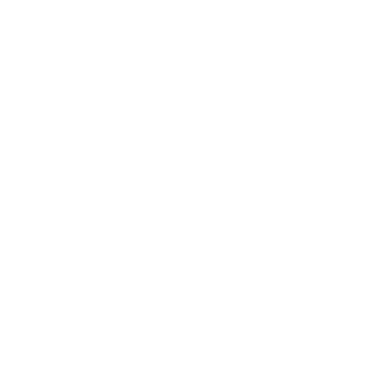
 Not at all.

13. How often does alopecia make it difficult for you to perform everyday activities (work, shopping, etc.)?


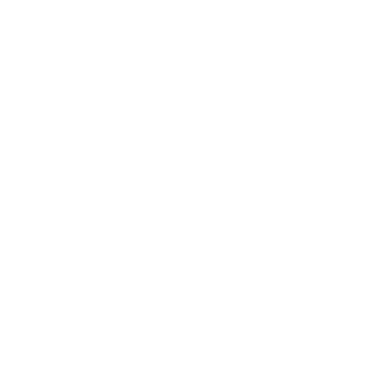
 Frequently;
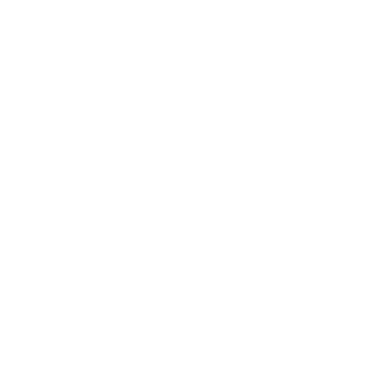
 Occasionally;
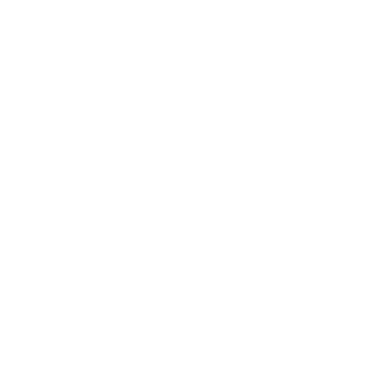
 Rarely;
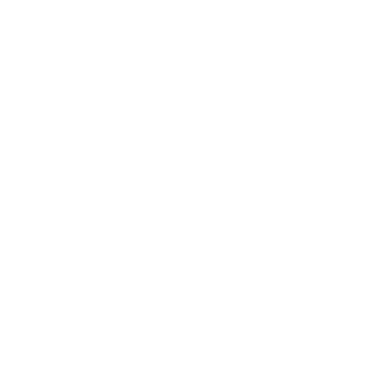
 Never;
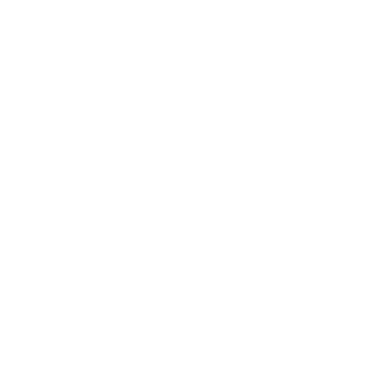
 Undecided.

14. How often does alopecia affect your social life / leisure activities?


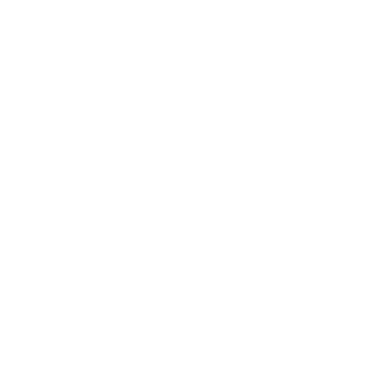
 Frequently;
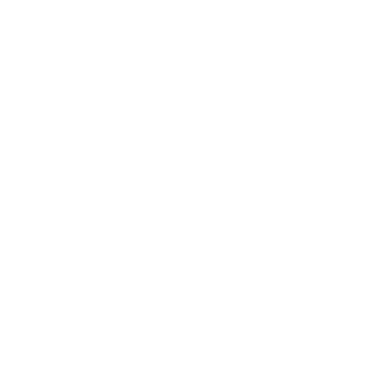
 Occasionally;
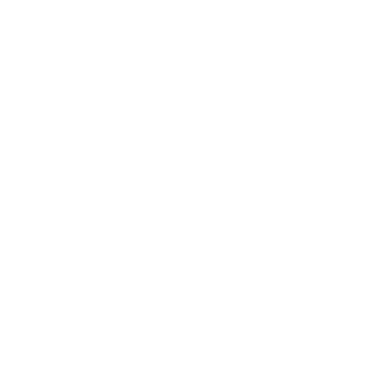
 Rarely;
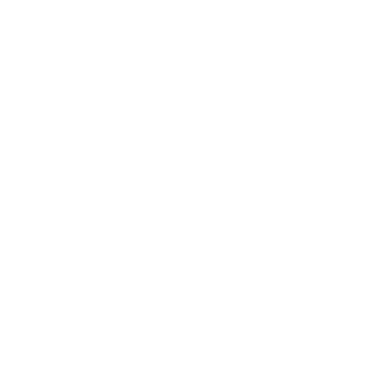
 Never;
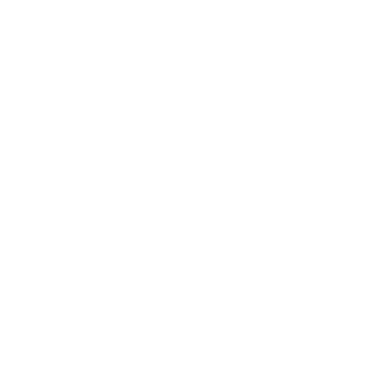
 Undecided.

15. To what extent is alopecia a problem in contact with a partner?


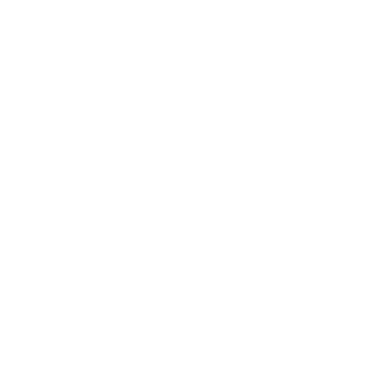
 To a great extent;
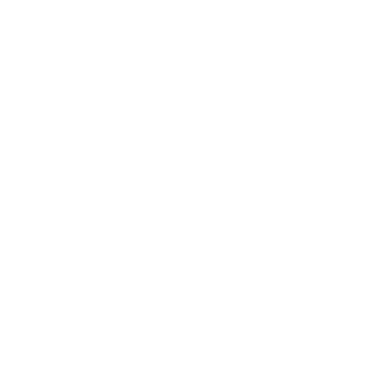
 Somewhat yes;
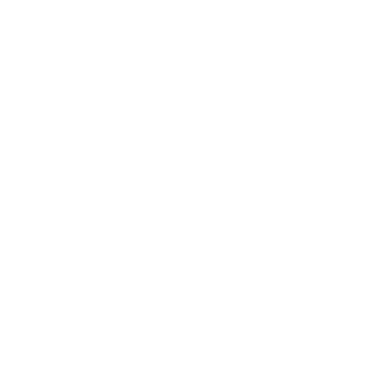
 Undecided;
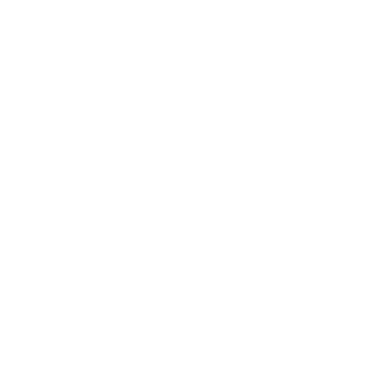
 Very little;
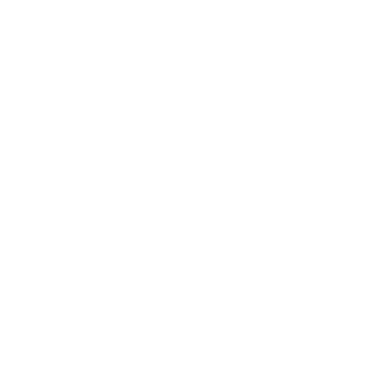
 Not at all.

16. Does alopecia affect the way you dress?


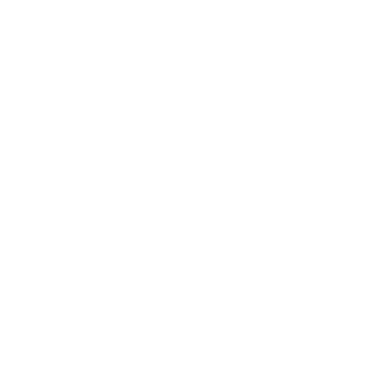
 To a great extent;
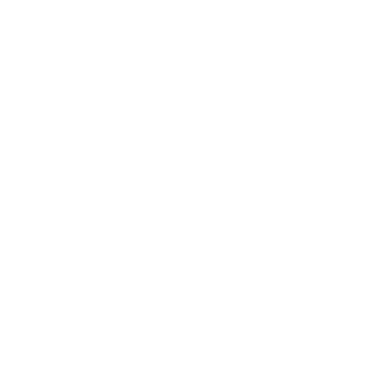
 Somewhat yes;
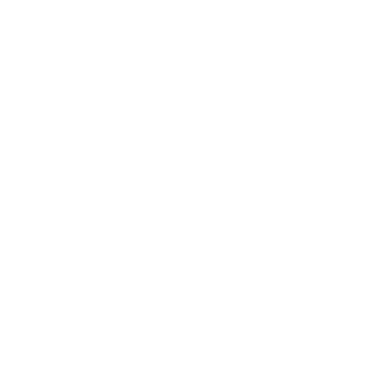
 Undecided;
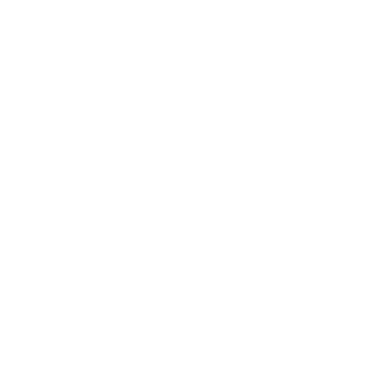
 Very little;
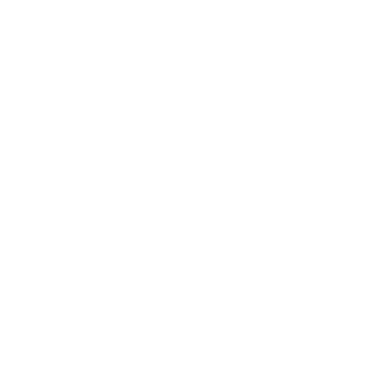
 Not at all.

17. Does alopecia prevent you from practicing sports in public places or at gyms?


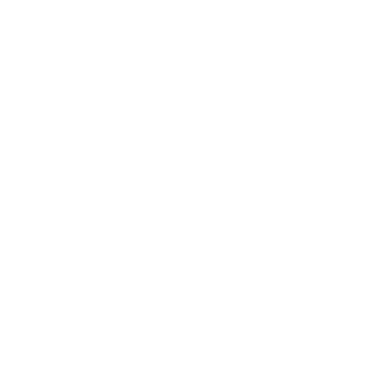
 To a great extent;
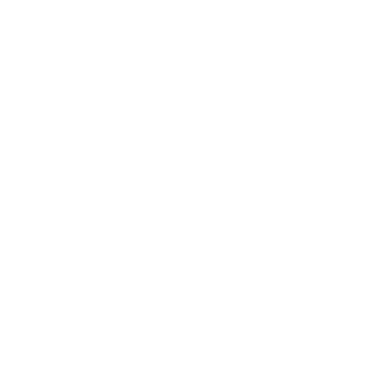
 Somewhat yes;
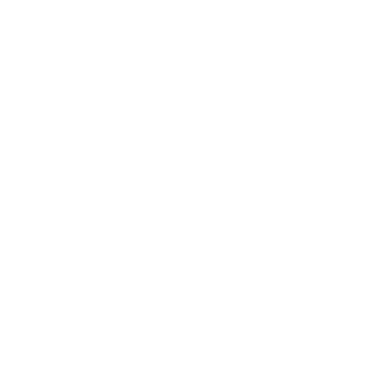
 Undecided;
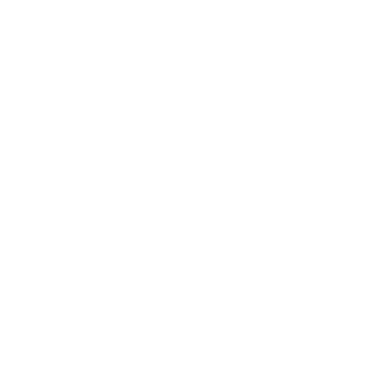
 Very little;
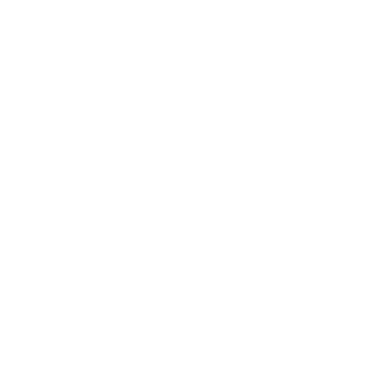
 Not at all.

18. How does excessive hair loss affect your self-esteem?


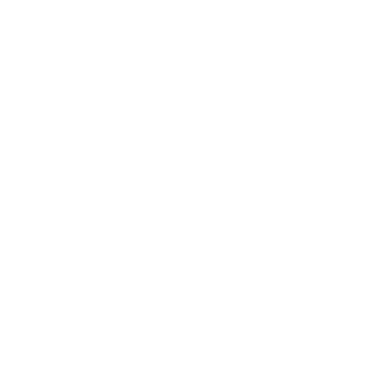
 To a Great Extent;
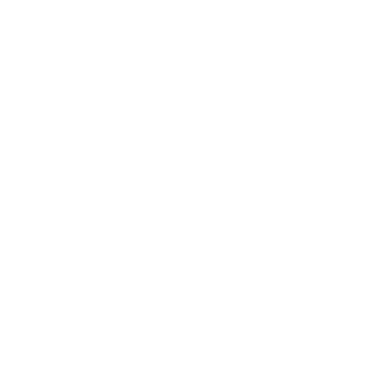
 Somewhat;
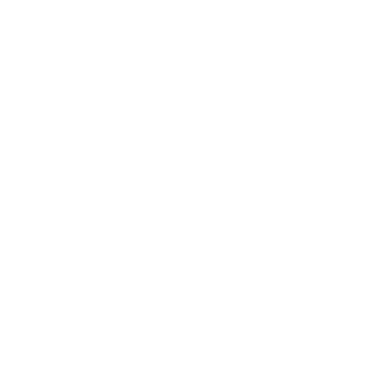
 Undecided;
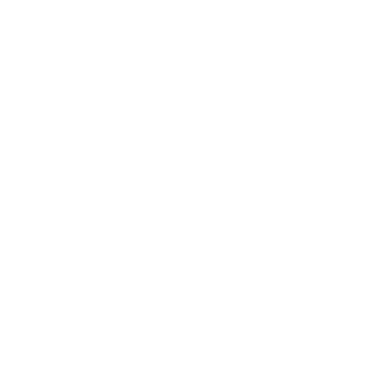
 Very Little;
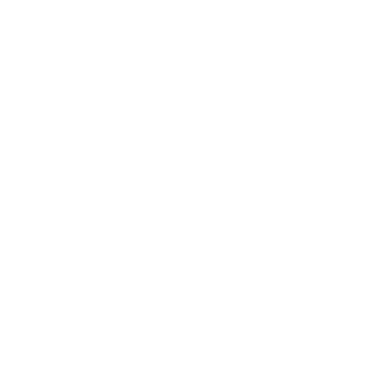
 Not at all.

19. How do jokes about baldness affect your self-esteem?


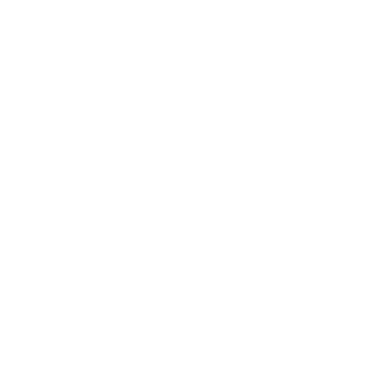
 To a Great Extent;
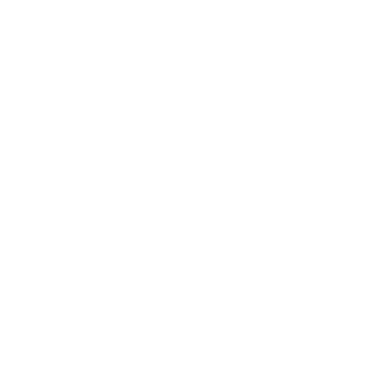
 Somewhat;
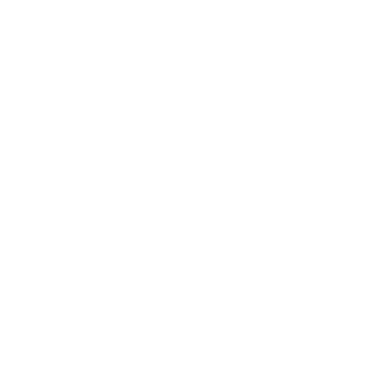
 Undecided;
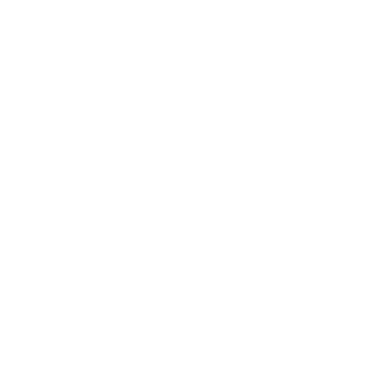
 Very Little;
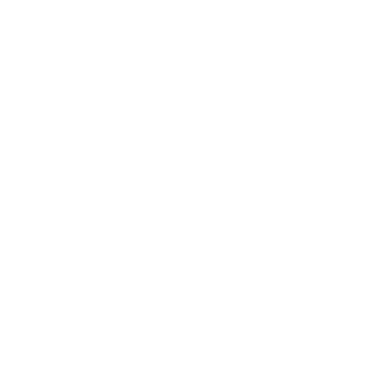
 Not at all.

20. How often do you think about your alopecia?


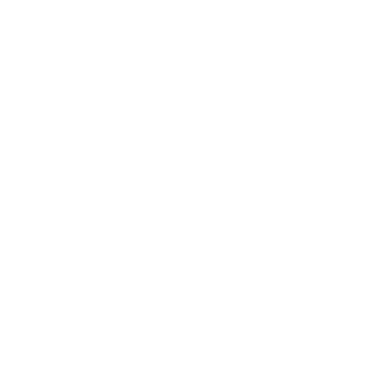
 Frequently;
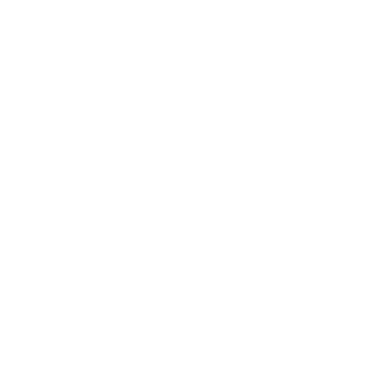
 Occasionally;
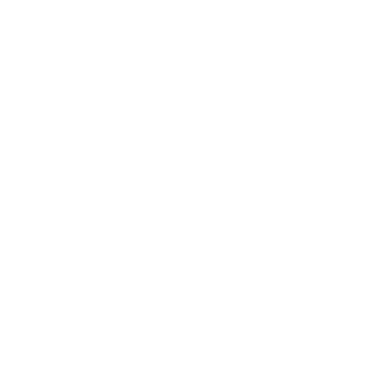
 Rarely;
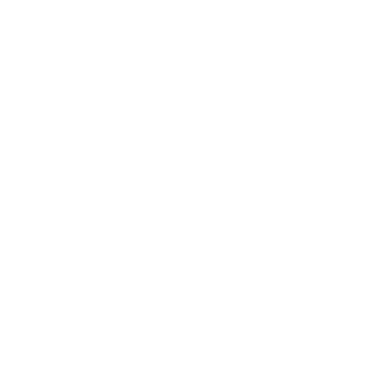
 Never;
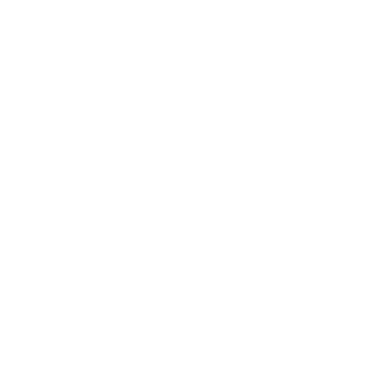
 Undecided.

21. In your opinion, are people suffering from androgenetic alopecia less attractive?


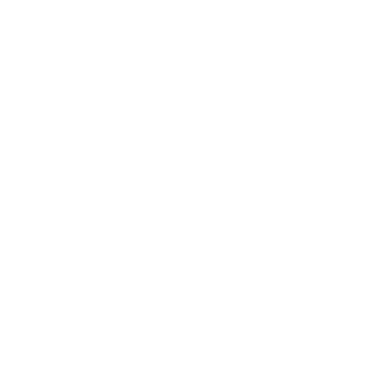
 To a great extent;
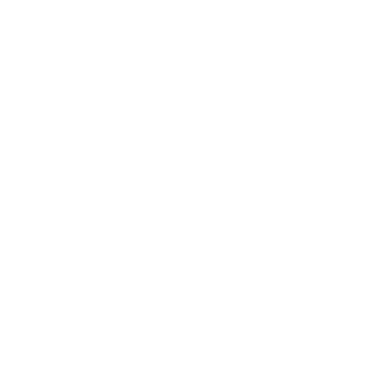
 Somewhat yes;
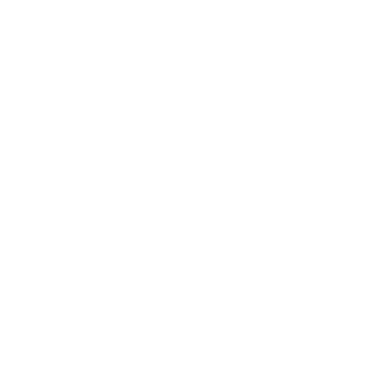
 Undecided;
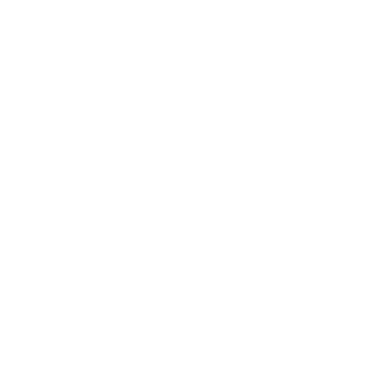
 Very little;
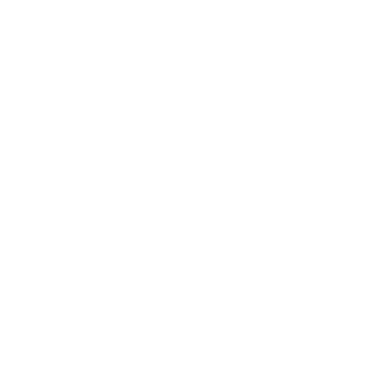
 Not at all.

22. Have you used any products or supplements to strengthen your hair or prevent hair loss?


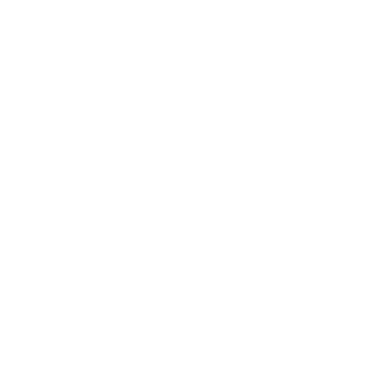
 Yes
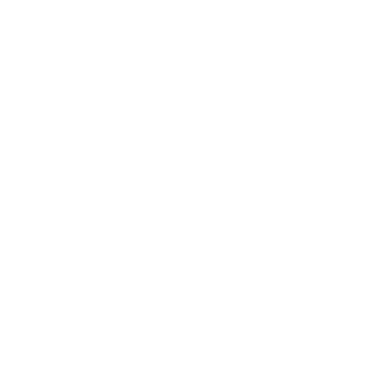
 No.

23. Have you undergone any treatments to prevent excessive hair loss?


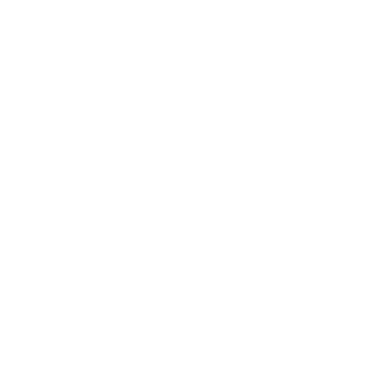
 Yes No.
